# TIM-3/Galectin-9 interaction and glutamine metabolism in AML cell lines, HL-60 and THP-1

**DOI:** 10.1186/s12885-024-11898-3

**Published:** 2024-01-24

**Authors:** Hooriyeh Shapourian, Mustafa Ghanadian, Nahid Eskandari, Abolfazl Shokouhi, Gülderen Yanikkaya Demirel, Alexandr V. Bazhin, Mazdak Ganjalikhani-Hakemi

**Affiliations:** 1 Department of Immunology, Faculty of Medicine, Isfahan University of Medical Sciences, Isfahan, Iran; 2 Department of Pharmacognosy, School of Pharmacy and Pharmaceutical Sciences, Isfahan University of Medical Sciences, Isfahan, Iran; 3 Department of Endocrine and metabolism research center, Isfahan University of Medical Sciences, Isfahan, Iran; 4 Department of Immunology, Faculty of Medicine, Yeditepe University, Istanbul, Turkey; 5 Department of General, Visceral and Transplant Surgery, Ludwig Maximilians University of Munich, Munich, Germany; 6 German Cancer Consortium (DKTK), Partner Site Munich, Munich, Germany; 7 Regenerative and Restorative Medicine Research Center (REMER), Research Institute for Health Sciences and Technologies (SABITA), Istanbul Medipol University, Istanbul, Turkey

**Keywords:** Acute myeloid leukemia (AML), T cell immunoglobulin and mucin-domain containing-3 (TIM-3), Galectin-9 (Gal-9), Glutamine metabolism, Glutaminase (GLS), Glutamate dehydrogenase (GDH)

## Abstract

**Background:**

T cell immunoglobulin and mucin-domain containing-3 (TIM-3) is a cell surface molecule that was first discovered on T cells. However, recent studies revealed that it is also highly expressed in acute myeloid leukemia (AML) cells and it is related to AML progression. As, Glutamine appears to play a prominent role in malignant tumor progression, especially in their myeloid group, therefore, in this study we aimed to evaluate the relation between TIM-3/Galectin-9 axis and glutamine metabolism in two types of AML cell lines, HL-60 and THP-1.

**Methods:**

Cell lines were cultured in RPMI 1640 which supplemented with 10% FBS and 1% antibiotics. 24, 48, and 72 h after addition of recombinant Galectin-9 (Gal-9), RT-qPCR analysis, RP-HPLC and gas chromatography techniques were performed to evaluate the expression of glutaminase (GLS), glutamate dehydrogenase (GDH) enzymes, concentration of metabolites; Glutamate (Glu) and alpha-ketoglutarate (α-KG) in glutaminolysis pathway, respectively. Western blotting and MTT assay were used to detect expression of mammalian target of rapamycin complex (mTORC) as signaling factor, GLS protein and cell proliferation rate, respectively.

**Results:**

The most mRNA expression of GLS and GDH in HL-60 cells was seen at 72 h after Gal-9 treatment (*p* = 0.001, *p* = 0.0001) and in THP-1 cell line was observed at 24 h after Gal-9 addition (*p* = 0.001, *p* = 0.0001). The most mTORC and GLS protein expression in HL-60 and THP-1 cells was observed at 72 and 24 h after Gal-9 treatment (*p* = 0.0001), respectively. MTT assay revealed that Gal-9 could promote cell proliferation rate in both cell lines (*p* = 0.001). Glu concentration in HL-60 and α-KG concentration in both HL-60 (*p* = 0.03) and THP-1 (*p* = 0.0001) cell lines had a decreasing trend. But, Glu concentration had an increasing trend in THP-1 cell line (*p* = 0.0001).

**Conclusion:**

Taken together, this study suggests TIM-3/Gal-9 interaction could promote glutamine metabolism in HL-60 and THP-1 cells and resulting in AML development.

**Supplementary Information:**

The online version contains supplementary material available at 10.1186/s12885-024-11898-3.

## Background

Acute myeloid leukemia (AML) is a type of hematological malignancy that manifests clonal cell proliferation, abnormal and poor differentiation of hematopoietic cells inside the bone marrow, blood, and other tissues [1, 2]. AML is the second most common type of leukemia diagnosed in children and adults, though most cases have been reported in adults [3]. While treatment outcomes have steadily improved in young adults over the past 20 years, there have been limited changes in survival for those over 60 years of age [4]. Therefore, there is a need to use new treatment strategies and reconsider past treatments.

Expression of T cell immunoglobulin and mucin-domain containing-3 (TIM-3) on malignant cells has been reported in some leukemia [5]. In most types of AML, especially HL-60 (the Myeloblastic (M2) subtype cell line) [6] and THP-1 (the Monocytic (M5) subtype cell line) [7], TIM-3 is expressed on leukemic stem cells (LSCs), but not on normal hematopoietic stem cells (HSCs) [8–10]. This protein can be a risk factor for poor prognosis, treatment response and also cause relapse in AML patients [11]. Consequently, targeting TIM-3 with an antibody, microRNA or other TIM-3 inhibitors eliminates LSCs while leaving normal HSCs unaffected [8, 9, 11]. Nowadays, MBG453 is the only monoclonal antibody that has demonstrated efficacy and early safety for myelodysplastic syndromes (MDS) and AML in clinical studies [12]. Furthermore, TIM-3 inhibition may lead to different outcomes in AML compared to other leukemia. Some clinical trials have shown that TIM-3 blockade alone fails to achieve clinical efficacy for most patients with AML or MDS and improvement in clinical outcome is only observed when TIM-3 is combined with other checkpoint inhibitors, such as tyrosine kinase inhibitors (TKIs) or hypomethylating agents (HMA) [11–13].

**Fig. 1:**
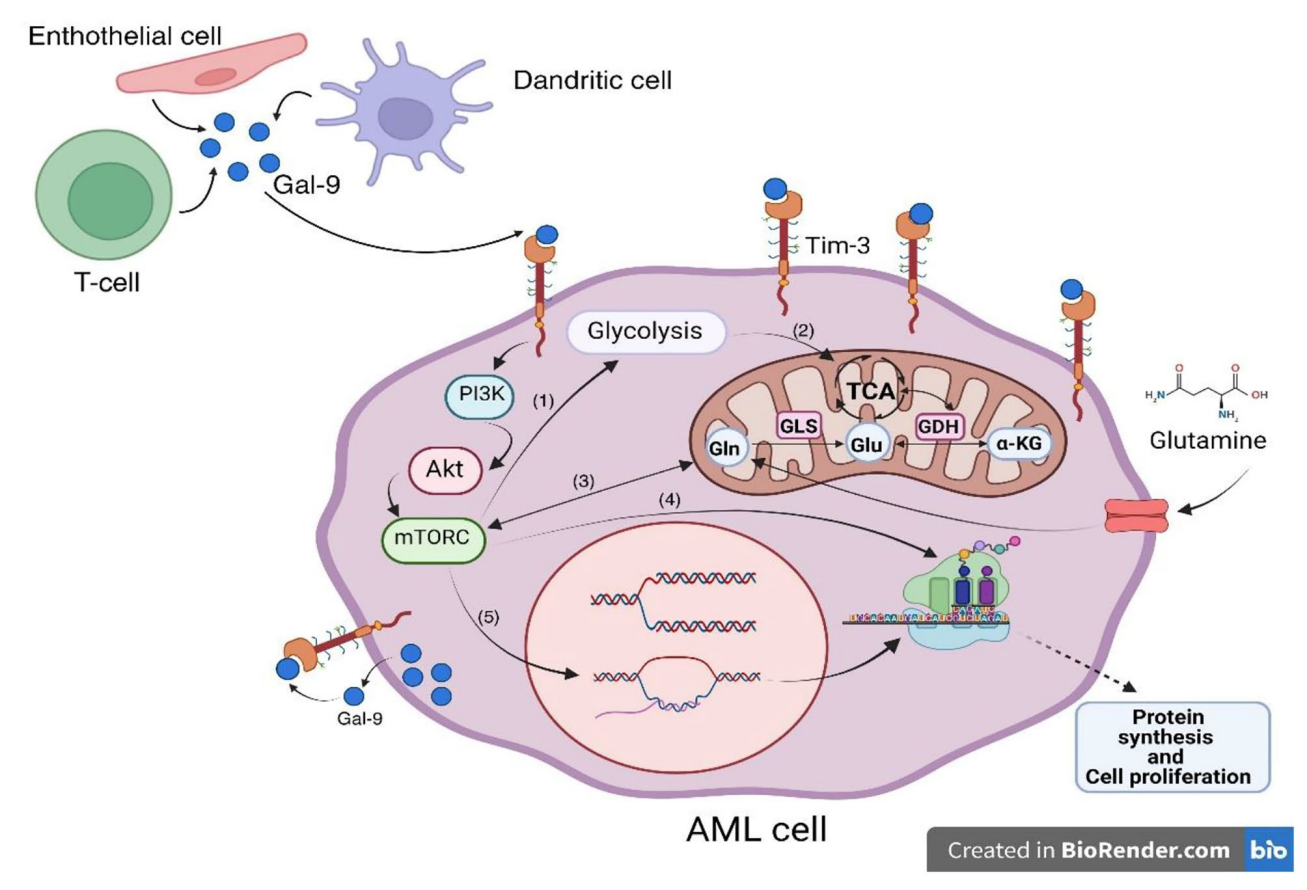
TIM-3/Gal-9 and glutamine metabolism. Gal-9 can be as free form or cell surface ligand on T cells, dendritic cells, and endothelial cells or other cells in the tumor microenvironment. After binding of the Gal-9 ligand to the TIM-3 receptor on the surface of AML leukemic cells through autocrine or paracrine pathways, the PI3K/AKT/mTORC signaling pathway is activated. mTORC is related to (1) the glycolysis pathway, (2) the Krebs cycle (TCA), (3) glutamine metabolism, (4) protein synthesis, and (5) cell proliferation. mTORC can act as an amino acid sensor and after the amino acid glutamine enters into the AML cell, it triggers (3) the pathway of glutamine degradation (glutaminolysis) by the enzymes GLS and GDH which respectively converts Gln to Glu and Glu to α-KG in mitochondria of leukemic blast. The dimensions of the cell components are relative and not real. The figure was Created in BioRender.com. TIM-3: T-cell immunoglobulin and mucin domain 3; Gal-9: Galectin-9; PI3k: phosphatidylinositol 3-kinase; AKT: or Protein kinase B (PKB); mTORC: mammalian target of rapamycin complex; TCA: tricarboxylic acid cycle; GLS: Glutaminase; GDH: Glutamate dehydrogenase; Gln; Glutamine; Glu: Glutamate; α-KG: alpha-ketoglutarate

As yet, four ligands have been found for TIM-3, Galectin-9 (Gal-9) is the most famous and first ligand that can induce apoptosis in Th1 cells and also stimulate other signaling pathways on different immune cells by binding to TIM-3 [11, 12, 14]. In general, galectins can bind to cell-surface molecules and extracellular matrix glycans on the outside of the cells and also, they may affect intracellular signaling pathways and protein–protein interactions in the cytosol and nucleus [14]. There is an autocrine stimulatory loop by interaction between TIM-3 and Gal-9 in AML cells that contributes to AML development and leukemic cell survival through phosphorylation of extracellular signal-regulated kinase (ERK), activation of protein kinase B (PKB, also known as AKT) and induction of the β-catenin pathway and nuclear factor kappa-light-chain-enhancer of activated B cells (NF-kB) [15–17]. On the other hand, the ligation of TIM-3 with Gal-9 can also stimulate the signaling pathways associated with metabolism, such as phosphatidylinositol-3 kinase (PI3K)/Akt/ mammalian target of rapamycin (mTORC) (Fig. 1). After mTORC1 activation, its substrates include the translation initiation factor 4E binding protein 1 (4EBP1) and ribosomal protein S6 kinase enhance protein synthesis and cellular proliferation [18–20]. Furthermore, mTORC1 increases intracellular calcium mobilization and upregulates the hypoxia-induced factor 1 (HIF-1) transcription complex and its downstream processes such as glycolysis and vascular endothelial growth factor (VEGF) production [21]. Together, these processes support cellular adaptation to pro-leukemic, inflammatory stress [20] and the promotion of amino acids metabolism [22]. Increasing glycolysis metabolites enhances the tricarboxylic acid cycle (TCA) and α-ketoglutarate which is associated with the metabolism of amino acids such as glutamate and glutamine (Fig. 1) [22].

As proteins are an important source for the immune cells metabolism, an imbalance between the consumption of amino acids by cancer cells and their deficiency for cellular immunity leads to cancer cells escape from the immune system [23]. Some amino acids specifically “glutamine” appear to play a prominent role in immunomodulation, proliferation, and activation of T cells and also malignant tumor progression, especially in their myeloid group [23–25]. Glutamine, the most abundant amino acid in the human body, is the second most important nutrient for cell proliferation, after glucose [25, 26]. Glutamine metabolism is very closely connected to mTORC1, a master controller of cell growth and metabolism [27]. After glutamine entrance into the mitochondria of neoplastic cells, the glutamine metabolic pathway (glutaminolysis) is initiated with the glutaminase (GLS) enzyme which converts glutamine (Gln) to glutamate (Glu) and subsequently, Glutamate dehydrogenase (GDH) alters Glu to alpha-ketoglutarate (α-KG) (Fig. 1) [28, 29]. This step is necessary for Gln to enter the TCA cycle. Studies revealed that GLS is overexpressed in cancer cells, especially in hematologic malignancies and AML [23, 24]. Consequently, in several leukemic cell lines, glutamine depletion can cause significant apoptosis [25].

In this research, we hypothesized that the interaction of TIM-3 with Gal-9 on HL-60 and THP-1 cells can be related to the glutamine metabolic pathway through the mTORC signaling route and AML progression. As a result of this study, we may be able to understand more aspects of the role of TIM-3 in glutamine metabolism in AML LSCs as well as go through the way of proposing potential therapeutic approaches in the future.

## Materials and methods

### Cell Culture

HL-60 (RRID:CVCL_0002) and THP-1 (RRID:CVCL_0006) cell lines (Paramedical Faculty of Shiraz, Iran) were cultured in Roswell Park Memorial Institute (RPMI) 1640 (BIO-IDEA, Iran) supplemented with 10% Fetal Bovine Serum (FBS; BIO-IDEA, Iran) and 1% antibiotics (100 U/ml penicillin and 100 µg/ml streptomycin) (denazist, Iran) [30, 31]. The cells were maintained at 37 °C in a humid atmosphere containing 5% CO2. After cell culturing, cells were divided into three groups: the first group was the control group which were cells without any treatment. The second group was the PMA group which 50 ng/ml of phorbol 12-myristate 13-acetate (PMA) was added to cells to stimulate more TIM-3 expression on cells [32]. The last group was PMA + Gal-9 (PG) group that after 24 h of treatment with PMA cells were washed with Phosphate-buffered saline (PBS) and then were treated with 100 ng/mL of human recombinant galectin-9 (Gal-9) (Biolegend, Germany) for 24 h (PG24), 48 h (PG48) and 72 h (PG72).

### RNA extraction, cDNA synthesis, and quantitative RT-PCR

After treatment, total RNA was extracted from each group through a parstous RNA extraction kit according to the manufacturer’s instruction (Parstous Inst, Iran) [33]. AddScript cDNA Synthesis Kit (AddBio, Korea) was used to synthesize the cDNA [34]. Real-time quantitative PCR (RT-qPCR) was performed on the transcripts by using a Step One Plus™ real-time DNA amplification system (Applied Biosystem, USA) [35] and 2X Real-Time PCR Master Mix, High ROX (SYBR Green I) kit (Ampliqon, Denmark), along with specific primers from Zist Fanavari Pishgam Inst, Iran. Specific primers for TIM-3, GLS, GDH, and β-actin (ACTB), as a housekeeping gene, were designed by Allele ID 7.0 software, and their sequences are listed in Table 1. A thermal cycling procedure was performed at one time at 95 °C for 15 min (denaturation for starting step) followed by 40 cycles of 95 °C for 15 s (Denaturation), 60 °C for 30 s (Annealing), and 72 °C for 45 s (Extension) [36]. The mRNA expression level of each gene was calculated in comparison to ACTB mRNA expression. The ${2}^{-\varDelta \varDelta Ct }$was used to calculate the relative expression of each studied gene.

**Table 1:** Primer sequences

No.	Gene symbol	Forward Primer Seq.	Reverse Primer Seq.
1	TIM-3	TGCTGCCGGATCCAAATC	GGTTTGATGACCAACTTCAGGTTAA
2	GLS	GAAGAAAGTTTGTGATTCCTGAC	AGTAGAATGCCTCTGTCCATC
3	GDH	ACTGATGTGAGTGTAGATGAAG	AATGCCAGGACCAATAAAGC
4	ACTB	TTCGAGCAAGAGATGGCCA	CACAGGACTCCATGCCCAG

### Flow cytometry

HL-60 and THP-1 cells from controls and PMA treated groups after 24 h treatment were collected from the wells and centrifuged. Then, the supernatant was removed and 2 µL of FITC anti-human TIM-3 (CD 366) (Biolegend, UK) was added to each cell pellet and samples were incubated at 4 °C for 30 min in the dark. Flow cytometer FACS Callibuor instrument (BD Bioscience, San Jose, USA) was used to measure the TIM-3 expression on the cells [31]. Results were analyzed by using Flowjo v10.5.3 software.

### Protein extraction, SDS-PAGE, and western blotting

GLS and mTORC protein expression was determined by western blot technique and compared to β-actin as loading control. Total protein from each group was extracted by using a protein extraction kit (Anacelltec Inst, Iran) according to the kit protocol. To measure the protein concentration in the samples, we used the Bradford test [37]. The optical absorbance (OD) of each sample was read at a wavelength of 595 nm with the Microplate reader (Hyperion, Germany). Protein samples with the amount of 50 µg/well were separated by 10% Sodium dodecyl-sulfate polyacrylamide gel electrophoresis (SDS–PAGE) and transferred onto 0.2 μm immune-Blot™ polyvinylidene difluoride (PVDF) membranes. The membranes were then blocked with 5% BSA in 0.1% Tween 20 for 1 h. Then, the membranes were incubated with Anti-mTORC, Anti-Glutaminase (Anti-GLS), and anti-β-actin-loading control antibodies (Abcam, United Kingdom) for 1 h at room temperature. Subsequently, membranes were washed thrice with Tris-buffered saline with 0.1% Tween 20 detergent (TBST), and incubated with goat anti-rabbit IgG H&L (HRP) (Abcam, United Kingdom) secondary antibody. Then, enhanced chemiluminescence (ECL) for 1–2 min was used to incubate the membranes. Protein expressions were normalized to β-actin. Densitometry of protein bands was performed by using the ImageJ Version 1.44 software and gel analyzer Version 2010a software (NIH, USA) [38, 39].

### MTT assay

For cell proliferation rate measuring, MTT DNA Biotech kit (kalazist Inst, Iran) was used. According to the kit protocol, 50,000 cells/well from each group (control, P24, PG72) were seeded duplicated in 96 well plates. In the first two wells, an equal amount with the cell volume of the culture medium without any cell was added as a blank solution. Based on the procedure, 10 µL of MTT reagent solution containing tetrazolium bromide and 90 µL of sterile PBS were added to each well. Then, cells were incubated in a 37 °C incubator for 3–4 h. After the incubation time, 100 µL of detergent solution was added to each well to dissolve the ferromazan crystals. In the last step, the optical density (OD) of each sample against blank was read at a wavelength of 570 nm with a microplate reader (Hyperion, Germany) [21]. This value was proportional to the number of living cells in each well.

### Reverse phase high-performance liquid chromatography (RP-HPLC)

For sample derivatization, 200 µL sample (cell culture supernatant of HL-60 or THP-1) + 50 µL internal standard (norleucine) + 800 µL methanol 10% were added to each other and centrifuged at 5000 rpm for 5 min. To derivatize, the supernatant was added to 100 µL MPA (100 µL 3-mercaptopropionic acid + 10 mL distilled water), 50 µL OPA (300 µL methanol + 20 mg ortho-phthalaldehyde + 10 mL distilled water) solutions [40], and 50 µL mobile-phase A (1 g potassium dihydrogen phosphate and 0.5 g dipotassium hydrogen phosphate in 500 mL distilled water). The resulting mixture was injected into HPLC after 2 min at room temperature. Reverse phase HPLC (RP-HPLC) was done on an Azura Knauer pump (Germany) and a fluorescence detector (Shimadzu Prominence RF-20 A, Japan) in 350 nm excitation/450 nm emission wavelength criteria by gradient elution on a C18 column (150 * 4.6 mm, 5 μm, Macherey-Nagel, Germany), using Mobile-phase A (1 g K2PO4 + 0.5 g KH2PO4 in 500 mL distilled water), and mobile- phase B (100 mL distilled water, 150 mL acetonitrile, and 300 mL isopropanol) [41, 42]. Gradient elution was linear from 10 to 100% B at a flow rate of 1.2 mL/min in 35 min. Before analysis different concentration of glutamate and glutamine standards (Sigma Aldrich, UK) was processed and derivatized in the same manner for sample, and injected into the HPLC for preparation of standard curve. The chromatograms were processed using Clarity software version 5.

### Gas chromatography (GC)

This technique was performed to measure α-KG concentration in each HL-60 and THP-1 cell culture supernatant. A Freeze dryer (National Appliance Company, Germany) was used to lyophilize control, PMA, and Gal-9 groups cell culture supernatants of each cell line. Then 500 µl of methanol was added to each of the dried samples and were dissolved well. Afterward, samples were centrifuged for 5 min at 4000 rpm. Extraction was performed twice and each time the supernatant was collected and completely dried by a Zymark TurboVap LV (Hopkinton, MA, USA) for 2 hours. Before GC analysis, the samples were made volatile by silyl reagents. For silyl derivatization, 50 µl of N,O-Bis(trimethylsilyl) trifluoroacetamide + trimethylchlorosilane (BSTFA + TMCS, 99:1) reagent was added to each sample [43] in the 2 mL GC closed vials and incubated for 60 min at 70 °C [44]. Then 1 µL of hexadecane (1 µg/mL) as an internal standard was added to the samples and standards. Finally, 1 µL of each sample was injected into the GC-FID system (GC-2550, Teif Gostar Faraz Co. Ltd., Iran) on a capillary column (TRB1, 30 m×0.25 mm, 1 μm) in a splitless mode. The carrier gas was hydrogen (11.2 psi pressure), with the column temperature program initiated at 100 °C (held for 3 min) to 250 °C @ 10 °C/min (held for 1 min). A standard curve was prepared by internal standard calibration using α-KG in different concentrations in addition to hexadecane (Sigma Aldrich, USA) as internal standard (1 µg/mL). The calibration curve was determined by linear regression in the range of 40-4000 µg/ml with the regression equation of y = 0.0009x + 0.7132, where X was the concentration of α-KG (µg/ml) with the R^2^ correlation factor of 0.9971.

## Statistical analysis

SPSS 27 and GraphPad Prism 9 software were performed for statistical analysis. Kolmogorov-Smirnov test has been applied to determine whether the distribution of the findings was normal. To compare results among the groups, one-way and two-way ANOVA tests were utilized. Results were calculated based on the means ± standard deviations (SD) of at least three experiments. *P* values of < 0.05 are considered significant.

## Results

### PMA increased TIM-3 expression on HL-60 and THP-1 cells

**Fig. 2:**
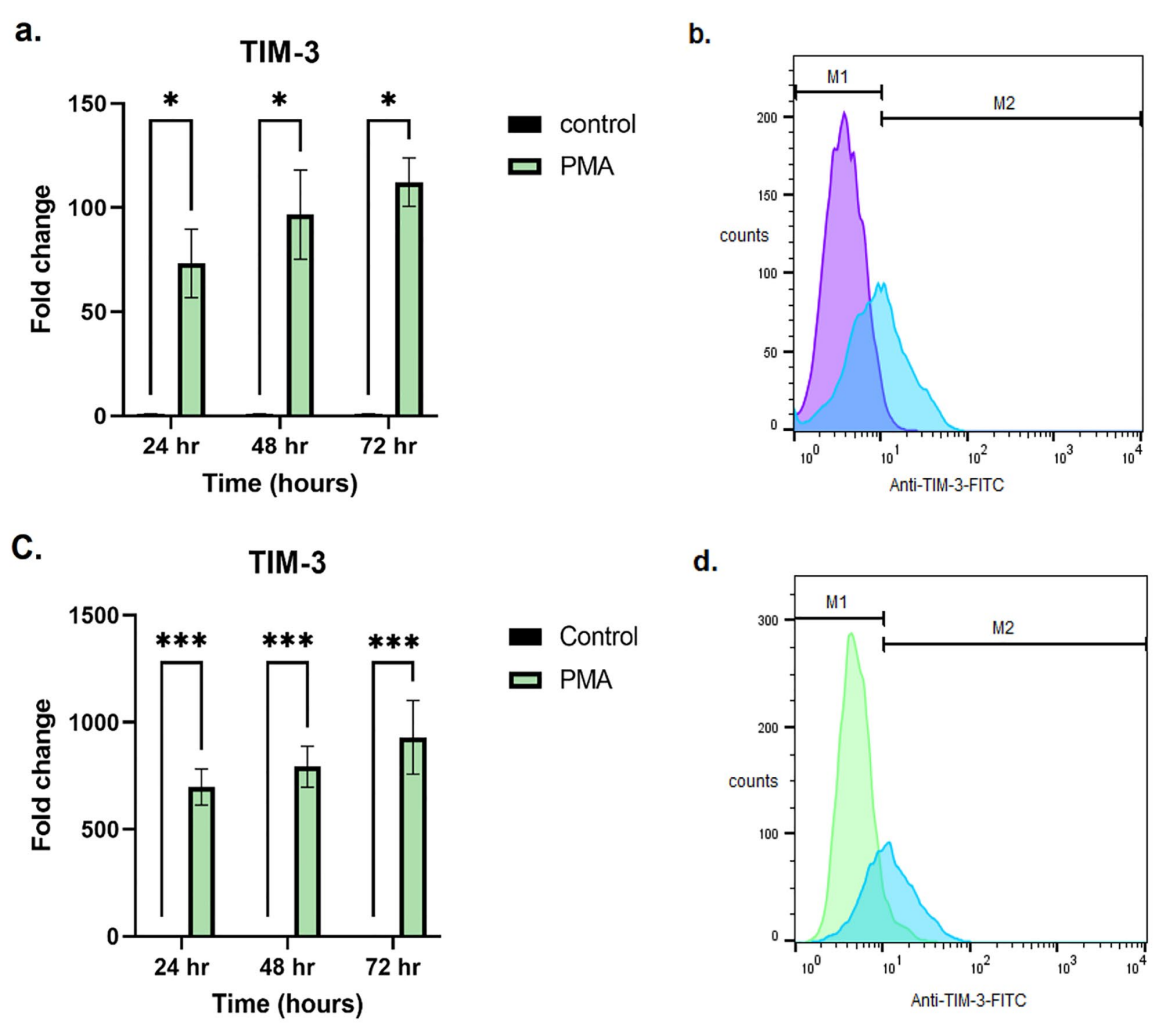
TIM-3 expression at 24, 48, 72 h after treatment with PMA in HL-60 and THP-1 cell lines. TIM-3 mRNA expression changes after stimulation with PMA compared to the control group in the HL-60 cell line (**a**) and THP-1 (**c.**) showed that a significant difference for TIM-3 expression at 72 h after PMA treatment was seen in the HL-60 cell line (*p* = 0.01) and THP-1 cell line (*p* = 0.0001). Asterisks represent statistical significance (* *p* < 0.05, ** *p* < 0.001, ***p < 0.0001). Histogram graphs of HL-60 cell line (**b**) before (purple curve) and after 24-hour treatment with PMA (blue curve) and THP-1 (**d.**) cell line before (green curve) and after 24-hour treatment with PMA (blue curve) revealed TIM-3 expression have increased to 47.7% and 63.9%, respectively

The mRNA expression level of TIM-3 in two cell lines had an increasing trend with a significant difference and the most expression level in comparison to the control group was at 72 h after PMA treatment in both HL-60 (mean = 112.21 ± 11.71, *p* = 0.01) and in THP-1 cell lines (mean = 929.30 ± 172.96, *p* = 0.0001) (Fig. 2 (a., c.)). Flow cytometry analysis showed that TIM-3 expression on the HL-60 cell line increased from 2.6% up to 47.7% and on THP-1 cells enhanced from 6.6% up to 63.9% after stimulation with PMA for 24 h (Fig. 2 (b., d.)).

### TIM-3/Gal-9 interaction increased GLS and GDH mRNA expression in the HL-60 cell line in a time course

**Fig. 3:**
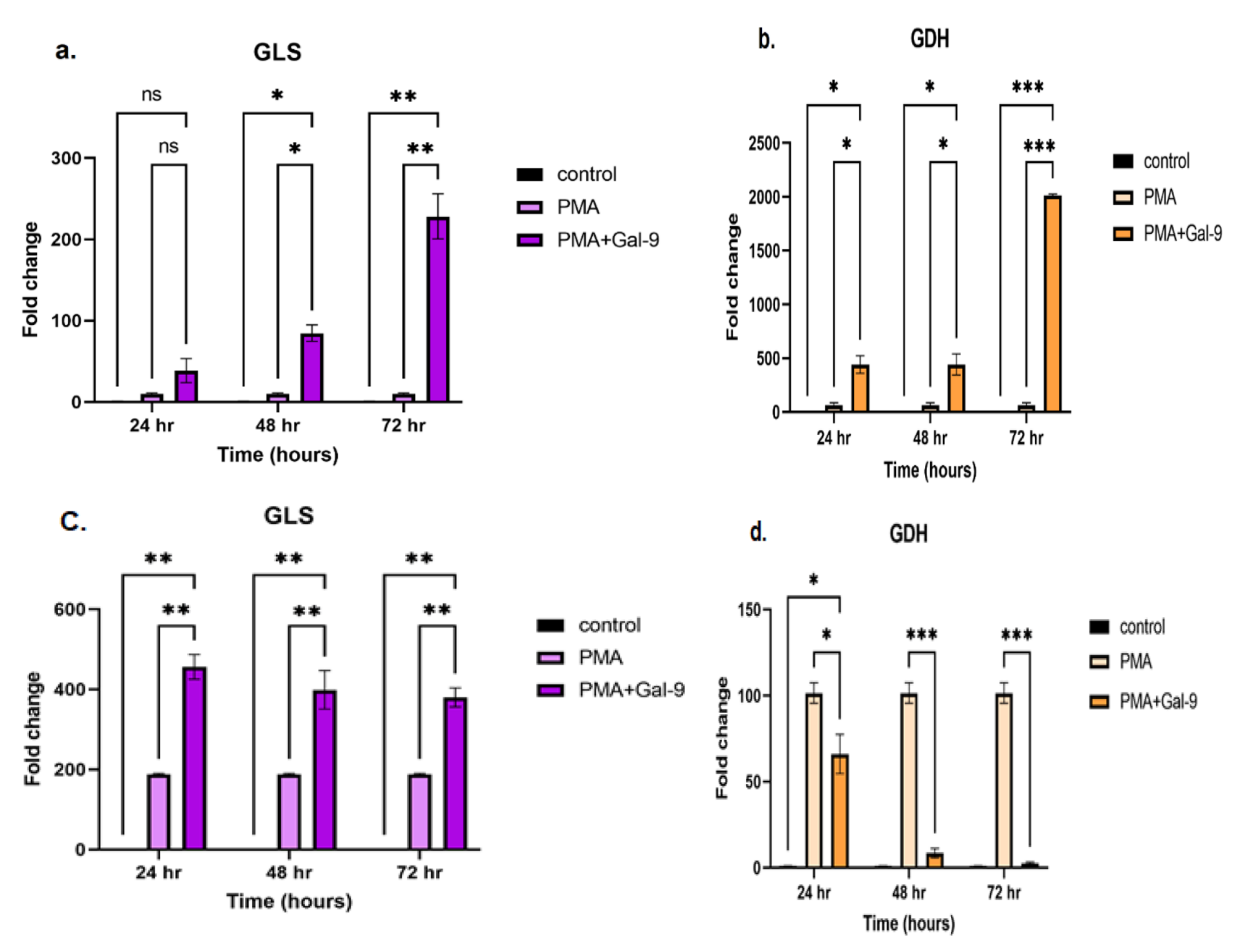
GLS and GDH mRNA expressions at 24, 48, 72 h after treatment of HL-60 and THP-1 cell lines with Gal-9. In HL-60 cell line, the expression of GLS enzyme at 24 h had no significant variation, but after 48 h (*p* = 0.01) and 72 h (*p* = 0.001), it was increased significantly in comparison to the control group (**a.**). GDH mRNA expression changes in HL-60 cells showed a significant difference between the PMA + Gal-9 group and the control group at 24 and 48 h (*p* = 0.02) and the most significant difference was achieved at 72 h (*p* = 0.0001) (**b.**). The kinetics of GLS (**c.**) and GDH (**d.**) mRNA expression changes after PMA + Gal-9 treatment compared to PMA and control groups in the THP-1 cell line indicated that the expression of GDH decreased in the time course. However, the GLS enzyme showed a significant increase in all time with the highest pick at the 24th hour (*p* = 0.001). Asterisks represent statistical significance (* *p* < 0.05, ** *p* < 0.001, ***p < 0.0001)

The mRNA expression level of glutaminase enzyme (GLS) and Glutamate dehydrogenase (GDH) in the HL-60 cell line had an increasing trend. The most mRNA expression of GLS and GDH in this cell line was seen at 72 h post Gal-9 treatment compared to PMA and control group (mean = 228.33 ± 27.77, *p* = 0.001) and (mean = 2012.82 ± 13.02, *p* = 0.0001), respectively. A significant difference in GLS mRNA expression at 48 h post Gal-9 treatment was observed in comparison to PMA and control group (*p* = 0.01). The mRNA expression of GDH increased after 24 and 48 h post Gal-9 treatment in comparison to PMA and control group (*p* = 0.02) (Fig. 3 (a., b.)).

### TIM-3/Gal-9 interaction increased GLS at the 24 h but decreased GLS and GDH mRNA expression in the THP-1 cell line as time passed

The mRNA expression level of GLS in THP-1 cell line samples treated with PMA + Gal-9 compared to the PMA and control groups in a time course (24, 48, and 72 h) showed a significant increase at 24 h followed by a gradual slight decrease as time went (*p* = 0.001). Although the mRNA level for GDH was higher at 24 h after Gal-9 treatment, it was significantly lower than its transcript level induced by PMA alone (*p* = 0.03). A significant difference in GDH mRNA expression at 48 and 72 h of treatment with Gal-9 in comparison to the PMA group was observed (*p* = 0.0001) (Fig. 3 (c., d.)).

**Fig. 4:**
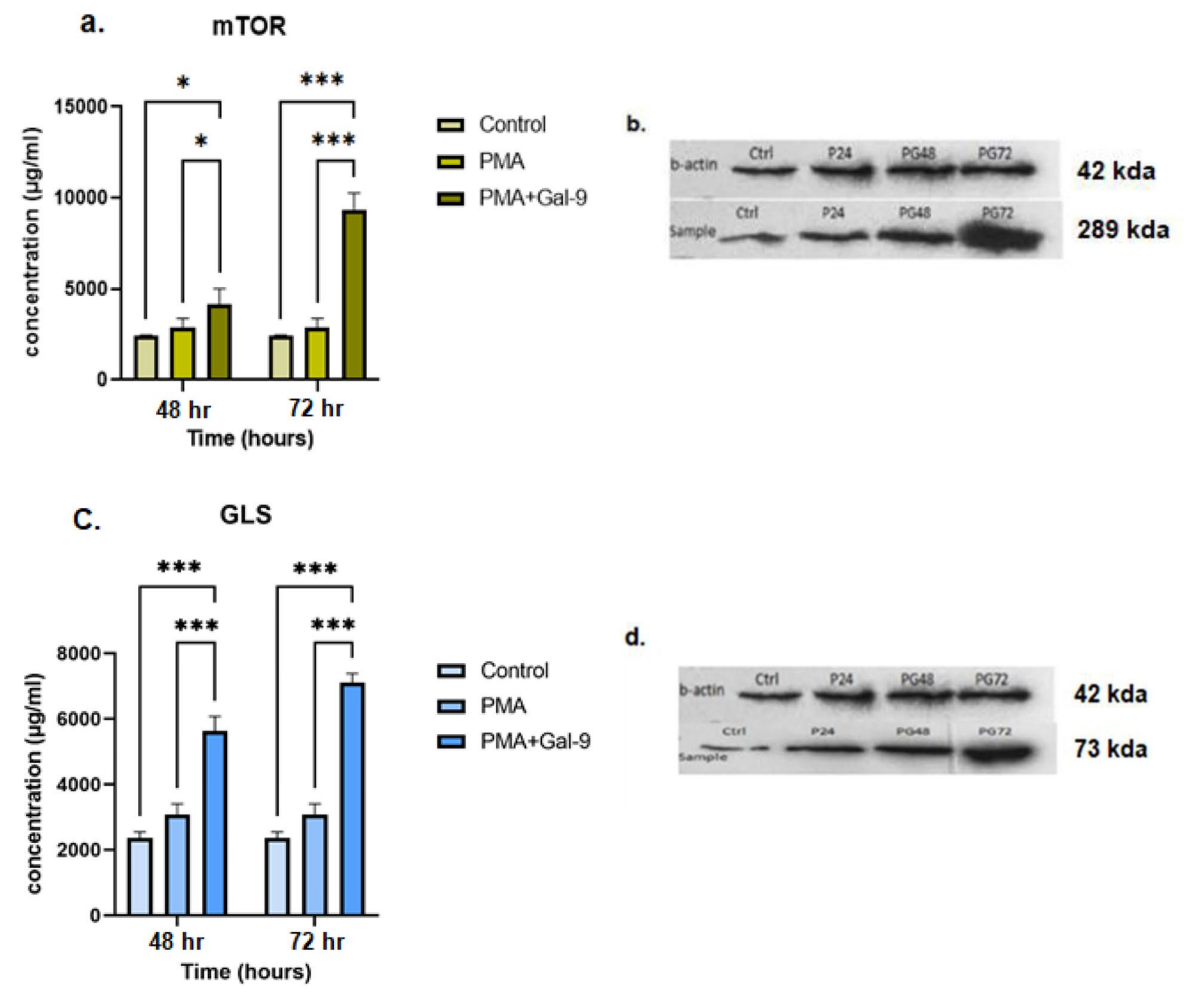
The mTORC and GLS proteins expression at 48 and 72 h after treatment with Gal-9 in the HL-60 cell line. Western blot analysis confirmed the results of RT-qPCR for mTORC and GLS protein expression in the HL-60 cell lines. Based on diagrams (**a.**, **c.**) and protein bands (**b.**, **d.**), the expression of these proteins in the Gal-9 treated group compared to the control and PMA group had an increasing trend with the highest level at 72 h after Gal-9 treatment (PG72) (*p* = 0.0001). Asterisks represent statistical significance (* *p* < 0.05, ** *p* < 0.001, ***p < 0.0001). Full-length blots/gels of b-actin, mTOR and GLS in the HL-60 cell line are presented in Supplementary Fig. 1 (**a.**), Fig. 2 (**a.**) and Fig. 3 (**a.**), respectively. The grouping of blots cropped from different parts of different gels

### TIM-3/Gal-9 interaction increased mTORC and GLS protein expression in the HL-60 Cell line in a time course

The results of western blot analysis for mTORC and GLS protein expression in the HL-60 cell line revealed that the expression of these proteins in the Gal-9 treated group compared to the control and PMA group had an increasing trend with a significant difference (*p* = 0.0001). The highest level of mTORC and GLS protein expression was observed at 72 h after Gal-9 treatment (*p* = 0.0001) (Fig. 4  (a., c.)). The expression level of mTORC protein in 48 h after treatment with Gal-9 showed a significant increase compared to PMA (4156 ± 847 (µg/ml), *p* = 0.04) and control (*p* = 0.01) group (Fig. 4  (a., b.)). A significant difference in GLS protein expression in the PG48 group compared to PMA and control group was perceived (*p* = 0.0001) (Fig. 4 (c., d.)).

**Fig. 5:**
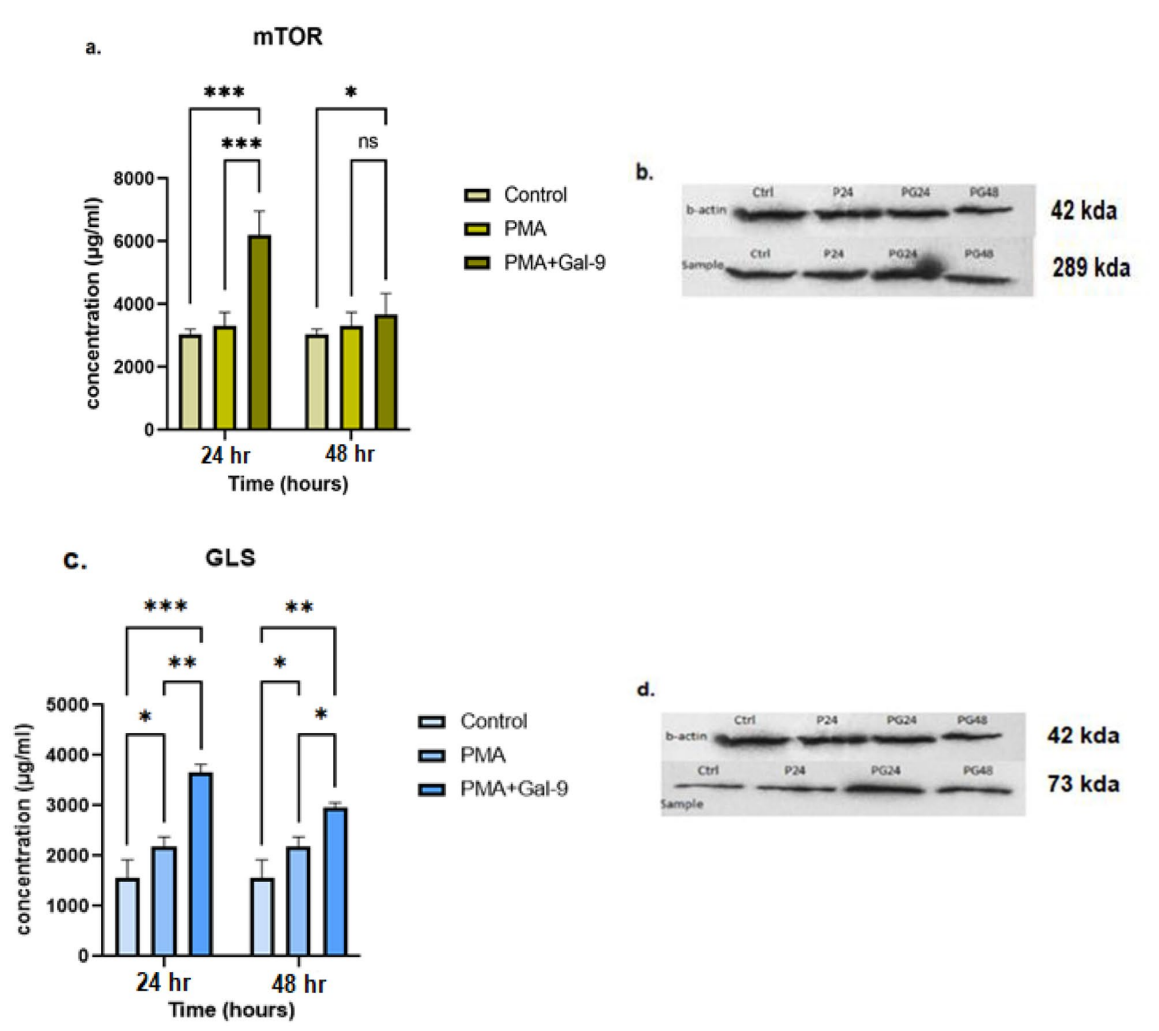
mTORC and GLS proteins expression at 24 and 48 h after treatment with Gal-9 in THP-1 cell line. Western blot analysis confirmed the results of RT-qPCR for mTORC and GLS protein expression in the THP-1 cell lines. Based on diagrams (**a.**, **c.**) and protein bands (**b.**, **d.**), the expression of these proteins in the Gal-9 treated group compared to the control and PMA group had a significant increase at 24 h after Gal-9 treatment (PG24) (*p* = 0.0001) followed by a decrease at 48th hour. Asterisks represent statistical significance (* *p* < 0.05, ** *p* < 0.001, ***p < 0.0001). Full-length blots/gels of b-actin, mTOR and GLS in the THP-1 cell line are presented in Supplementary Fig. 1 (**b.**), Fig. 2 (**b.**) and Fig. 3 (**b.**), respectively. The grouping of blots cropped from different parts of different gels

### TIM-3/Gal-9 interaction decreased mTORC and GLS proteins expression in the THP-1 cell line in a time course

The results of western blot analysis showed that the expression of mTORC and GLS proteins in the THP-1 cell line had a spike at 24 h after Gal-9 treatment (*p* = 0.0001). However, mTORC protein expression decreased significantly after 48 h (*p* = 0.04) (Fig. 5  (a., b.)). Although, a significant increase in GLS protein expression after 24 h post Gal-9 treatment was seen in comparison to the PMA group, this expression decreased after 48 h (*p* = 0.001, *p* = 0.01) (Fig. 5 (c., d.)).

**Fig. 6:**
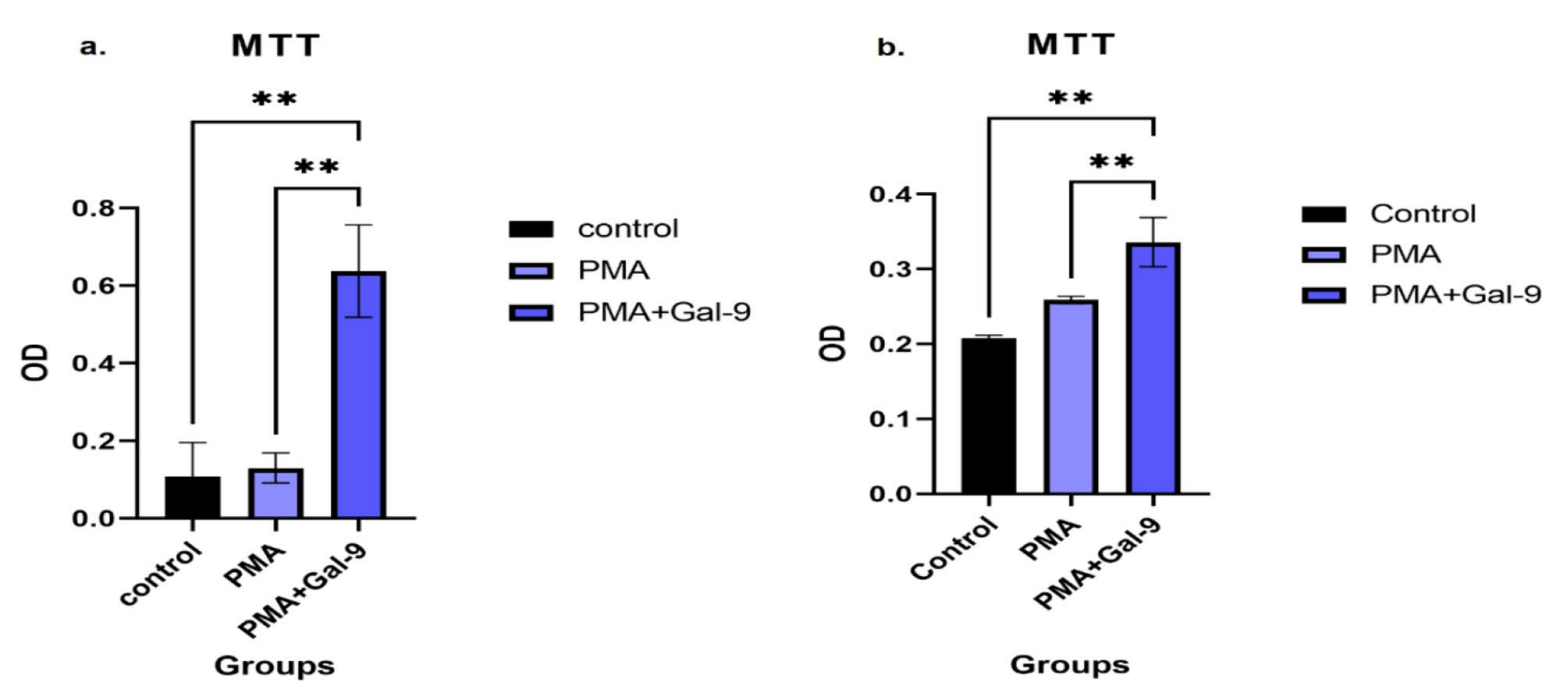
Cell proliferation rate changes in control, PMA, and PMA + Gal-9 groups for HL-60 and THP-1 cell lines. The kinetics of OD changes in the PMA + Gal-9 (PG72) group compared to PMA (P24) and control groups in the HL-60 cell line (**a**) and THP-1 (**b**) showed that treatment with Gal-9 significantly increases the proliferation and survival of the AML cell lines. Asterisks represent statistical significance (* *p* < 0.05, ** *p* < 0.001, and *** *p* < 0.0001)

### TIM-3/Gal-9 interaction promoted cell proliferation rate in both AML cell lines

MTT assay was implemented to investigate the cell proliferation rate in each group. Our results indicated that the cell proliferation rate was increased in the PMA + Gal-9 group in comparison to the PMA and control group (*p* = 0.001) in both HL-60 and THP-1 cell lines (Fig. 6 (a., b.)).

**Fig. 7:**
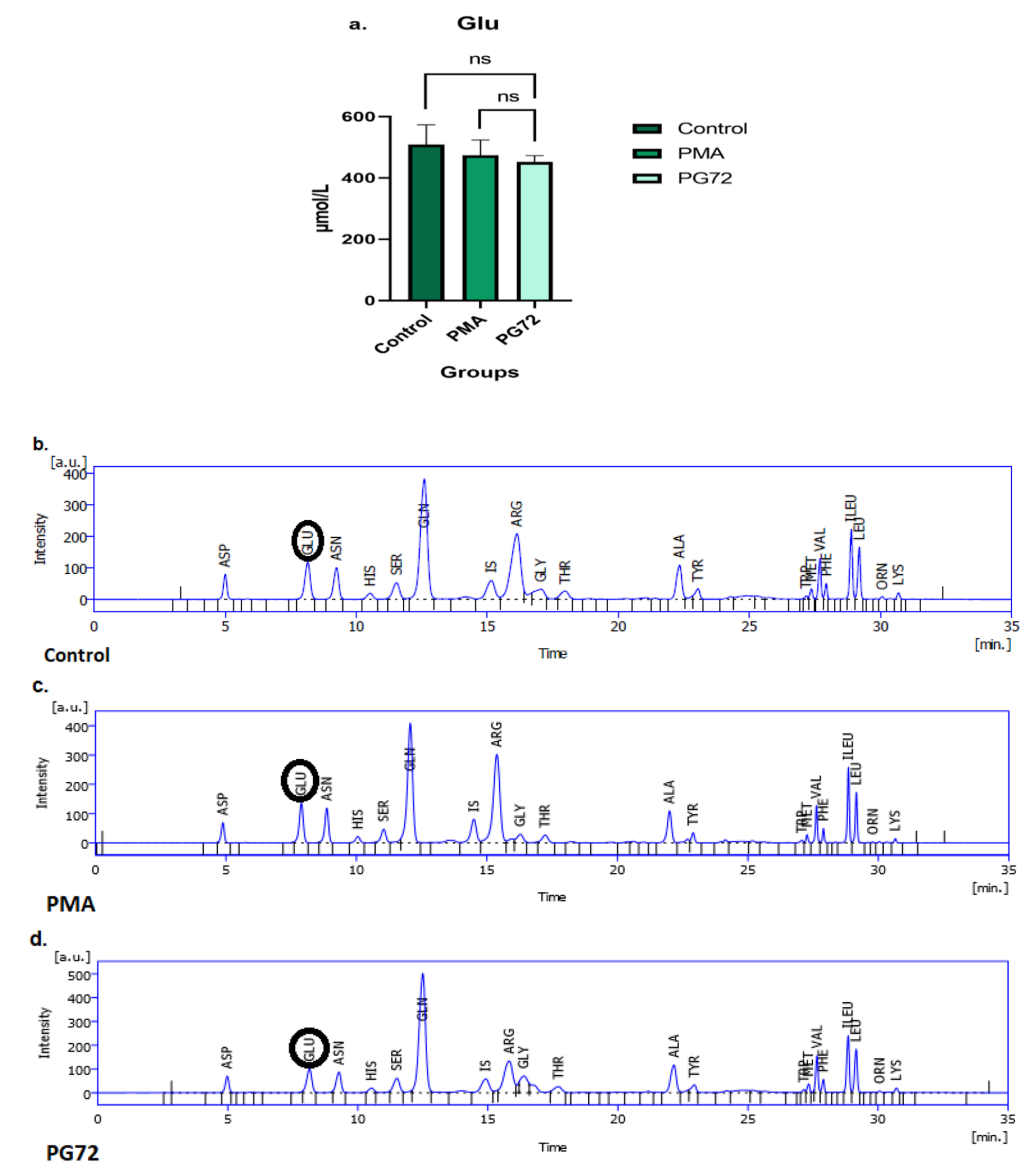
Changes of glutamate level in control, PMA, and PG72 samples of HL-60 cell line and RP-HPLC chromatograms. The trend of glutamate concentration after treatment with galectin-9 in the HL-60 cell line was decreasing in comparison to the PMA and control group but with no significant difference. (**a.**) Displays changes in glutamate concentration (µmol/L) in control, PMA, and PG72 groups in the HL-60 cell line. (**b.**), (**c.**), and (**d.**) Represents glutamate (GLU) in retention time of 8.15 min, 7.89 min, and 8.14 min for control, PMA, and PG72 groups, respectively. RP-HPLC method, OPA/MPA precolumn derivatization using fluorescence detector excitation 350 nm and emission 450 nm wavelengths. RP-HPLC was done on a C18 column (150 * 4.6 mm, 5 μm), using Mobile-phase A (1 g K2PO4 + 0.5 g KH2PO4 in 500 mL distilled water), and B (100 mL distilled water, 150 mL acetonitrile, and 300 mL isopropanol) with linear gradient from 10–100% B at a flow rate of 1.2 mL/min in 35 min

### Glutamate level after TIM-3/Gal-9 interaction in HL-60 and THP-1 cell lines

**Fig. 8:**
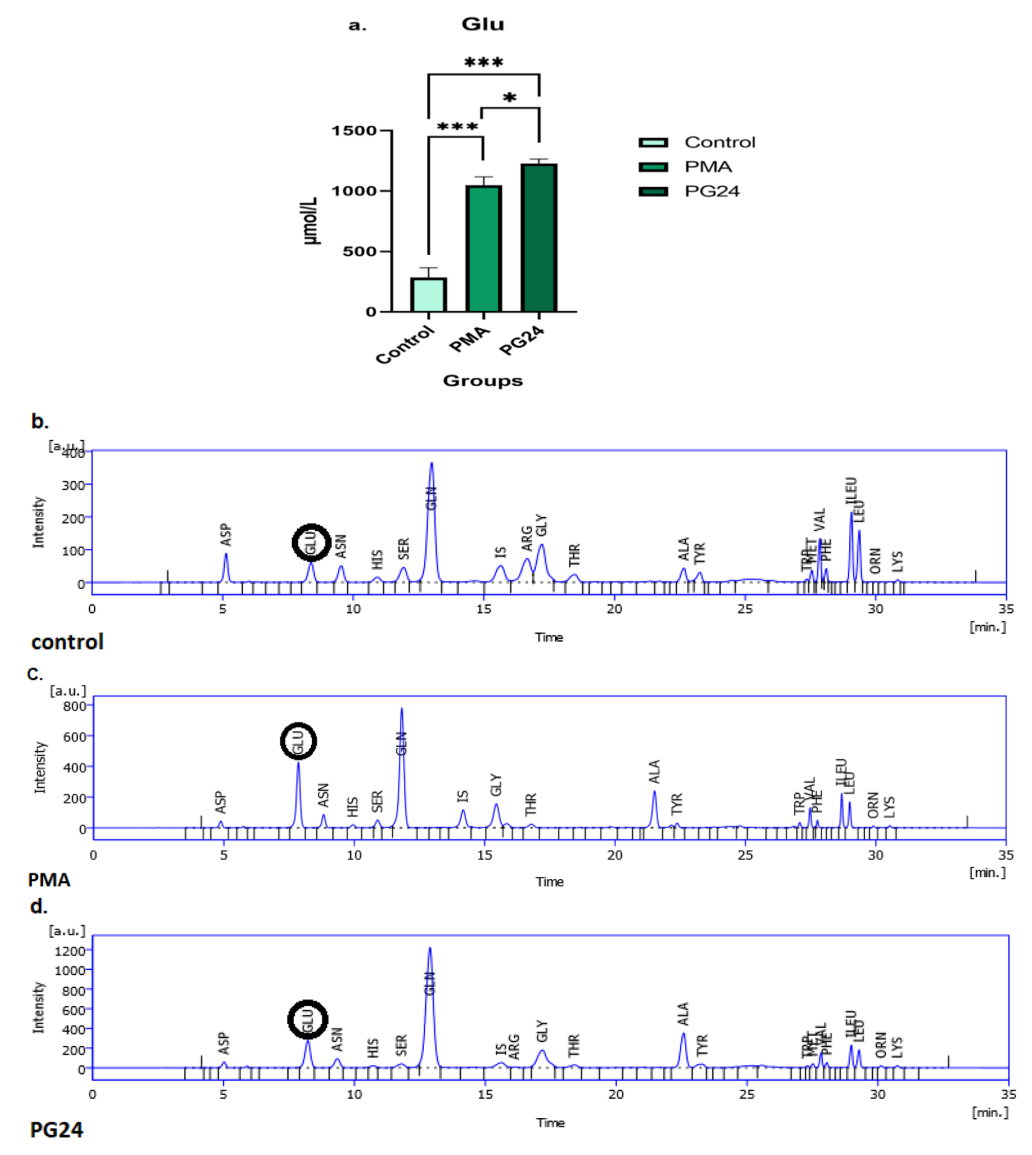
Changes of glutamate level in control, PMA, and PG24 groups of THP-1 cell line with RP-HPLC chromatograms. The trend of glutamate concentration after treatment with galectin-9 in the THP-1 cell line was increasing in comparison to the PMA and control group. The most Glu concentration was at 24 h after Gal-9 treatment (*p* = 0.0001) (* *p* < 0.05, ** *p* < 0.001, ***p < 0.0001). (**a.**) Displays changes in glutamate concentration (µmol/L) in control, PMA, and PG24 groups in the THP-1 cell line. (**b.**), (**c.**), and (**d.**) Represents glutamate (GLU) in retention time of 8.38 min, 7.85 min, and 8.23 min for control, PMA, and PG24 groups, respectively. RP-HPLC Method, OPA/MPA precolumn derivatization using fluorescence detector excitation 350 nm and emission 450 nm wavelengths. RP-HPLC was done on a C18 column (150 * 4.6 mm, 5 μm), using Mobile-phase A (1 g K2PO4 + 0.5 g KH2PO4 in 500 mL distilled water), and B (100 mL distilled water, 150 mL acetonitrile, and 300 mL isopropanol) with linear gradient from 10–100% B at a flow rate of 1.2 mL/min in 35 min

The results of the RP-HPLC technique for the determination of glutamate (Glu) metabolite in samples have revealed that the concentration of glutamate decreased in the HL-60 cell line after 72 h of treatment with Gal-9 compared to the PMA and control group, even though this reduction was not significant statistically (*p* = 0.37) (Fig. 7). Our results for the THP-1 cell line indicated that the concentration of Glu enhanced after 24 h of treatment with Gal-9 compared to the PMA and control group. A significant increase in Glu concentration in the PMA group compared to the control group was seen (*p* = 0.0001) and for the PG24 group compared to PMA and control group was perceived (*p* = 0.03) and (*p* = 0.0001), respectively (Fig. 8).

**Fig. 9:**
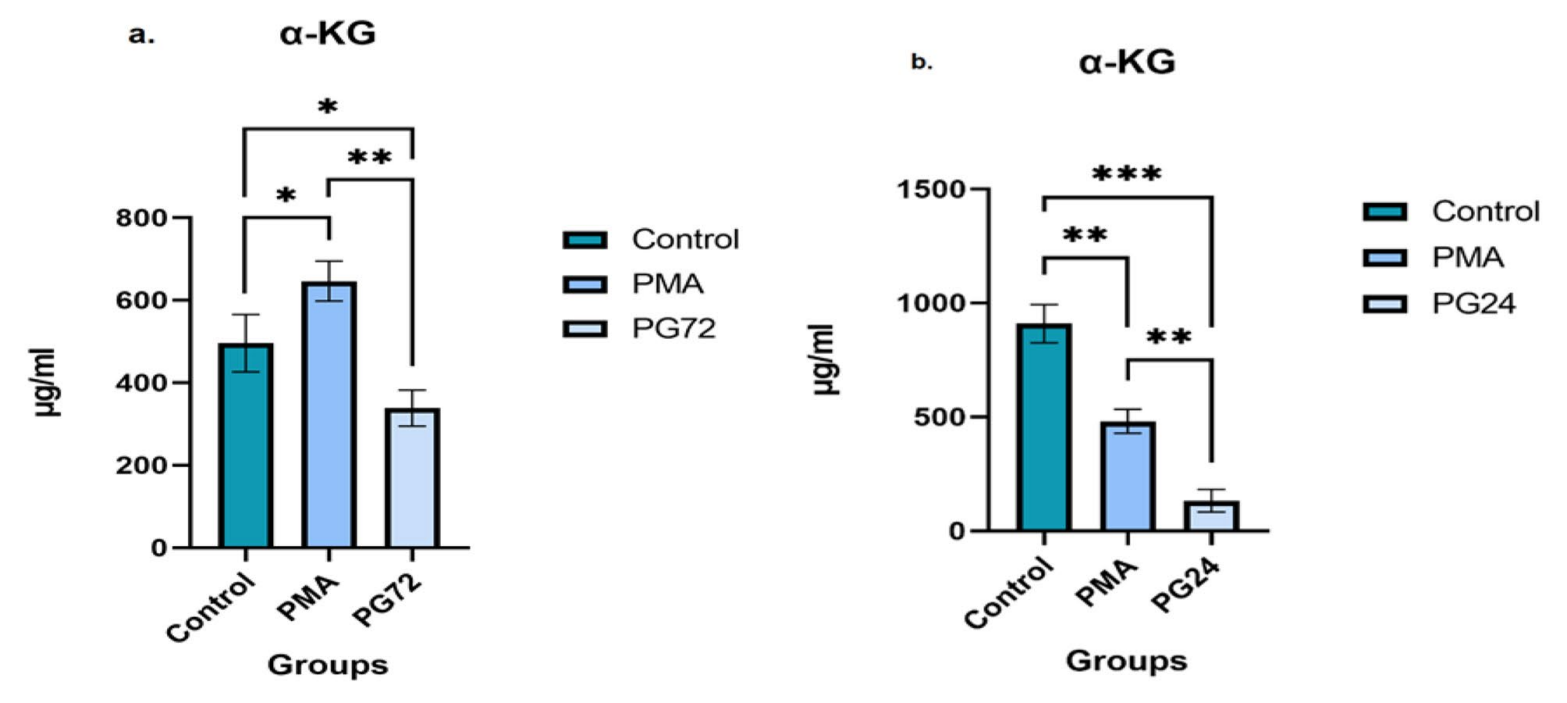
Changes of α-KG level in control, PMA, and PG72 samples of HL-60 and THP-1 cell lines. The trend of α-KG concentration after 72 h treatment with galectin-9 in the HL-60 (**a**) and 24 h treatment with gal-9 in THP-1 (**b**) cell lines was decreasing in comparison to PMA (*p* = 0.001, *p* = 0.001) and control (*p* = 0.03, *p* = 0.0001) groups, respectively. (* *p* < 0.05, ** *p* < 0.001, ***p < 0.0001)

### Alpha-ketoglutarate (α-KG) level was reduced after TIM-3/Gal-9 interaction in both HL-60 and THP-1 cell lines

Evaluation of the keto acid alpha-ketoglutarate (α-KG) in the supernatants of HL-60 cell cultures by gas chromatography (GC) technique indicated a significant decrease in the concentration of α-KG post 72 h Gal-9 treatment compared to the control (*p* = 0.03) and PMA (*p* = 0.001) group. Significant variation for the PMA group compared to the control group was reported (*p* = 0.04) (Fig. 9 (a.)). The results of the GC for α-KG in the supernatants of THP-1 cell cultures showed that the concentration of this keto acid decreased after 24 h of treatment with Gal-9 compared to the control (*p* = 0.0001) and PMA (*p* = 0.001) groups. A significant difference was also observed for the PMA compared to the control group (*p* = 0.001) (Fig. 9 (b.)).

## Discussion

Acute myeloid leukemia (AML) is the most common type of leukemia in adults and still has the lowest survival rate of all leukemia [4]. We aimed to evaluate the relation between TIM-3/Gal-9 interaction and the pathway of glutamine metabolism in two AML cell lines, HL-60 and THP-1. HL-60 cells are capable of expressing high levels of TIM-3 when stimulated with phorbol myristate acetate (PMA) [9, 45]. Our RT-qPCR and flow cytometry results for TIM-3 expression confirmed this stimulation not only on HL-60 cells but also on THP-1 cells.

The TIM-3/Gal-9 interaction on leukemic cells can activate mTORC signaling factor which is correlated with glutamine metabolism and acts as an amino acid sensor in the cell [46]. Therefore, we measured the expression of mTORC protein before and after Gal-9 treatment, and the results showed that the expression level of this factor was increased significantly in each cell line (*p* = 0.0001) which happened sharply at 24 h in THP-1 but gradually and after 48 h in HL-60 cell line. Alexandr Prokhorov et al., have shown that the TIM-3 activation by galectin-9, triggers growth factor type responses by stimulation of the PI-3 K/mTOR pathway in AML cells especially in THP-1 cells and also VEGF production and induced S2448 mTOR phosphorylation were shown time-dependent [20]. In addition, another study indicated that as a result of glutamine removal, mTORC1 could be inhibited in AML cells. The findings have shown that the glutaminase activity of L-asparaginase (l-ase) from Escherichia coli and Erwinia chrysanthemi inhibits mTORC1, and protein synthesis, and induces apoptosis in primary AML cells, especially in HL-60 cell line [47]. It suggests the difference in the expression level and the time of the mTORC signaling factor increase could be due to the difference in binding affinity of galectin-9 to TIM-3 over time as well as time-dependent mTORC phosphorylation in THP-1 and HL-60 cell lines.

Our results showed that TIM-3/Gal-9 interaction increased GLS transcript and protein levels in both HL-60 and THP-1 cell lines. However, this increase in the HL-60 cell line was at 72 h, and for THP-1 cell line was at 24 h after Gal-9 addition. However, although the expression of GDH showed a significant increase in HL-60 cells after TIM-3/Gal-9 interaction, its level showed a significant decrease in THP-1 cells in the same condition. In line with our study, Mineaki Goto et al. revealed that in the AML cell lines examined (HL-60, THP-1, NB4, and Kasumi-1) glutamine was the most important nutrient for HL-60 cells, and HL-60 was the most sensitive cell line to glutamine deprivation and blocking glutaminolysis by oxaloacetic acid, an intermediate of TCA cycle. Furthermore, they showed that after the growth of cell lines in a medium with glycolysis inhibitor (2-deoxyglucose(2-DG)) for 24 h, HL-60 cells produced the most abundant NH4+, while THP-1 cells produced the least which illustrated the least glutamine dependency of THP-1 [48]. They have also demonstrated that a major component of THP-1 cells’ energy supply and metabolite production is mitochondrial oxidative phosphorylation, which involves in fatty acid oxidation [49–51]. Based on the glutaminolysis pathway, glutamine is metabolized by GLS, which liberates NH4 + to produce glutamate, which is then metabolized by GDH that liberates another molecule of NH4 + and generates α-KG. Thus, the more glutamine is used by the cell, the more NH4 + will be released. These findings determine that HL-60 has high glutamine dependency for providing energy and also the production of intermediates for macromolecule biosynthesis and TIM-3/Gal-9 interaction helps this cell line to achieve its requirements more convenient. Other metabolic pathways could be a priority in THP-1 cells to provide biomass energy. Also, it suggests that TIM-3/Gal-9 interaction is more correlated with other metabolic pathways in THP-1 cells than glutaminolysis and this interaction may promote other metabolic pathways like glycolysis, krebs cycle (TCA), oxidative phosphorylation and etcetera to produce consumable α-KG and providing energy in this cell line. So, in this way TIM-3/Gal-9 interaction reduces or inhibits expressions of glutaminolysis enzymes like; GDH that are less needed. GLS can act as a rate-limiting factor for the tricarboxylic acid (TCA) cycle and it can replenish this cycle through α-KG production [22]. In other words, the higher the TCA cycle activity we have, the greater the production of α-KG is in a cell, and the lower the activity of glutaminolysis pathway enzymes (GLS, GDH) is needed.

In the current study, glutamate (Glu) as a GLS product, was reduced in PMA and Gal-9 treated groups in comparison to the control group in the HL-60 cell line. Nevertheless, this metabolite had an increasing trend in the THP-1 cell line between groups. We also showed that the mRNA expression of the GDH enzyme was much higher than the mRNA expression of GLS in the HL-60 cell line. Therefore, we may probably conclude that although a large amount of glutamate was produced by GLS, GDH also consumed it, or even Glu may have been regenerated from other metabolic pathways such as transaminase activity with compensatory mechanisms [52]. So, there was no significant reduction of Glu between the HL-60 groups.

The results indicated that α-KG concentration reduced after Gal-9 addition in HL-60 and THP-1 cell lines. In the HL-60 cell line, first, the α-KG concentration increased after PMA treatment and then reduced after 72 h of Gal-9 addition. After treatment with PMA, cells are activated and proliferation increases, hence, cells need to provide energy and produce macromolecules to the intermediate compounds resulting from the decomposition of glutamine and glutamate like α-KG, so the amount of α-KG increases. The α-KG reduction in the PG72 group could be due to the decrease in the amount of glutamate in the treated groups for the production of non-essential amino acids or purine and pyrimidine nucleotides for DNA synthesis in the cells, as well as the activation of the cell and the Krebs cycle [29]. However, in the THP-1 cell line, due to the high expression of GLS enzyme and the decreasing trend of GDH after TIM-3/Gal-9 interaction, the increase of Glu amount and reduction of α-KG concentration was observed in treated groups.

Results of the MTT assay have shown that TIM-3/Gal-9 interaction could increase cell proliferation rate in both HL-60 and THP-1 cells in the PMA + Gal-9 group compared to the PMA and control group. In line with our study, Yoshikane Kikushige et al. in 2015 represented that expansion and transformation of malignant myeloid clones could happen through ligation of TIM-3/Gal-9 and induction of the NF-κB and β-catenin signaling pathway at the same time through its autocrine loop which caused in production of leukemia transformed models from MDS, Myeloproliferative neoplasms (MPN), and also in de novo AML [52].

## Conclusion and future recommendations

In conclusion, our study could indicate the importance of metabolic rewiring in hematological cancer therapy, especially in AML. Nowadays, it seems essential more than ever to perform in-vivo, preclinical, and clinical studies in the field of using combinational therapies that target both immunological and metabolic pathways, for example, TIM-3 and glutamine metabolism pathway in hematological malignancies such as AML.

Due to the lack of funds and the increase in the number of tests, we were not able to perform western blot and chromatography tests for the samples of all 24, 48 and 72 h after treatment with Gal-9. So, we recommend for future studies, in other times when these tests were not applied, perform the necessary tests to make a more accurate comparison between groups and times. It is suggested to evaluate the relationship between TIM-3/Gal-9 pathway and the metabolism of other important amino acids that play a role in the progression of AML disease (arginine and tryptophan, etc.) in the AML cell lines mentioned in the study. Also, evaluating the relationship of these pathways in peripheral blood or bone marrow samples taken from AML patients and measuring the protein expression of GDH enzyme with western blot technique are other suggestions of this research for future studies.

### Electronic supplementary material

Below is the link to the electronic supplementary material.

Supplementary Material 1

## Data Availability

Some of the data of this study is available in a supplementary file but, other datasets used and analysed during the current study are available from the corresponding author on a reasonable request.

## Abbreviations

TIM-3: T cell immunoglobulin and mucin-domain containing-3
AML: Acute myeloid leukemia
Gal-9: Galectin-9
GLS: Glutaminase
GDH: Glutamate dehydrogenase
mTORC: Mammalian target of rapamycin complex
Gln: Glutamine
Glu: Glutamate
α-KG: Alpha-ketoglutarate
LSCs: Leukemic stem cells
HSCs: Hematopoietic stem cells
MDS: Myelodysplastic syndromes
TKIs: Tyrosine kinase inhibitors
HMA: Hypomethylating agents
ERK: Extracellular signal-regulated kinase
PKB: Protein kinase B
NF-kB: Nuclear factor kappa-light-chain-enhancer of activated B cells
PI3K: Phosphatidylinositol-3 kinase
4EBP1: Initiation factor 4E binding protein 1
HIF-1: Hypoxia-induced factor 1
VEGF: Vascular endothelial growth factor
TCA: Tricarboxylic acid cycle
