# Supplementary material for: TIM-3/Galectin-9 interaction and glutamine metabolism in AML cell lines, HL-60 and THP-1
Source: BMC Cancer. 2024 Jan 24;24:125. doi: 10.1186/s12885-024-11898-3 (PMC10809689; doi:10.1186/s12885-024-11898-3)
Supplement: Supplementary file 1 — Supplementary Material 1 [file 12885_2024_11898_MOESM1_ESM.docx]

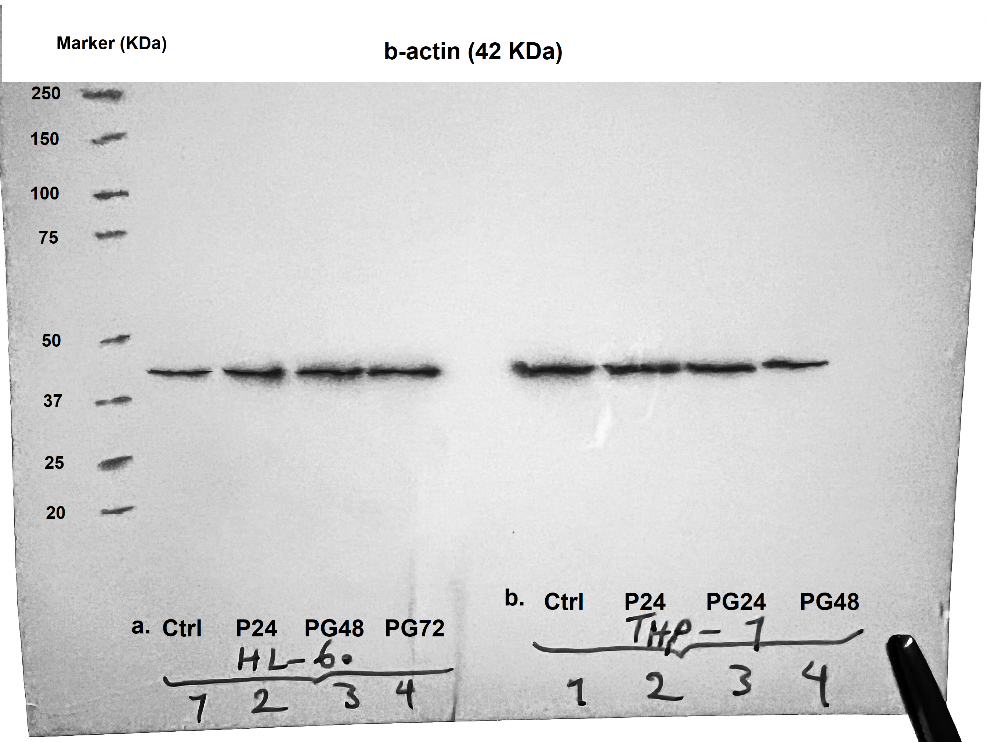


**Supplementary figure 1. The b-actin protein expression in HL-60 and THP-1 cell lines.** (a.) Displays the full-length gels of b-actin protein bands in control (ctrl), 24 hours after PMA treatment (P24), 48 hours (PG48) and 72 hours (PG72) after Gal-9 treatment groups in the HL-60 cell line. (b.) Presents the full-length gels of b-actin protein bands in control (ctrl), 24 hours after PMA treatment (P24), 24 hours (PG24) and 48 hours (PG48) after Gal-9 treatment groups in the THP-1 cell line. Cropped 4 grouping blots are displayed in the Figure 4 (b., d.) for the HL-60 cell line and Figure 5 (b., d.) for the THP-1 cell line in the manuscript, respectively.


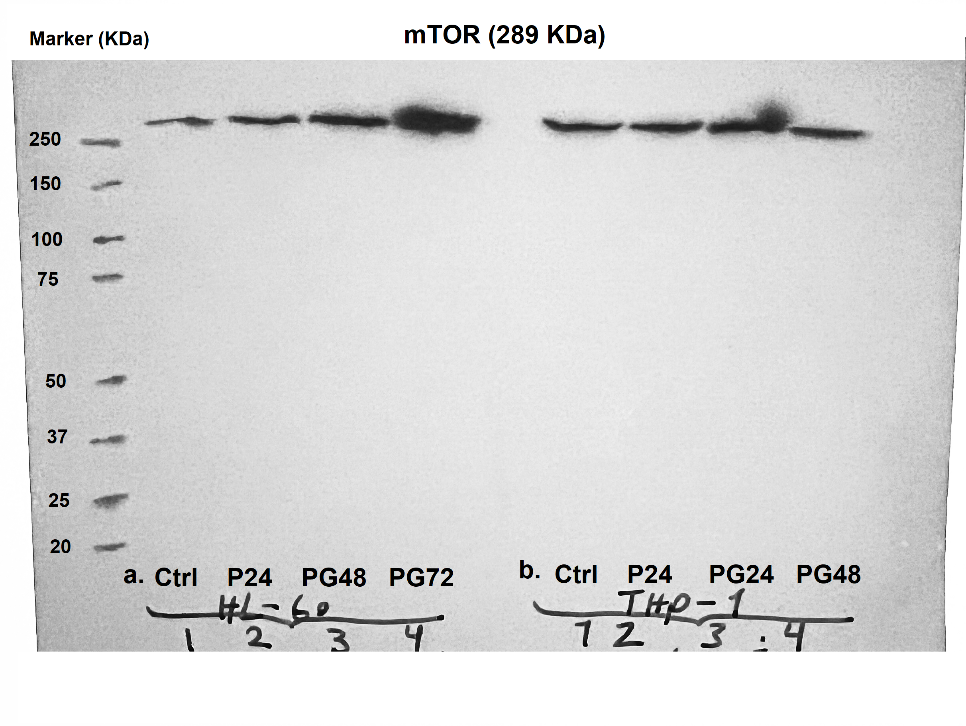


**Supplementary figure 2. The mTOR protein expression in HL-60 and THP-1 cell lines.** (a.) Displays the full-length gels of mTOR protein bands in control (ctrl), 24 hours after PMA treatment (P24), 48 hours (PG48) and 72 hours (PG72) after Gal-9 treatment groups in the HL-60 cell line. (b.) Presents the full-length gels of mTOR protein bands in control (ctrl), 24 hours after PMA treatment (P24), 24 hours (PG24) and 48 hours (PG48) after Gal-9 treatment groups in the THP-1 cell line. Cropped 4 grouping blots are displayed in the Figure 4 (b.) for the HL-60 cell line and Figure 5 (b.) in the THP-1 cell line in the manuscript, respectively.


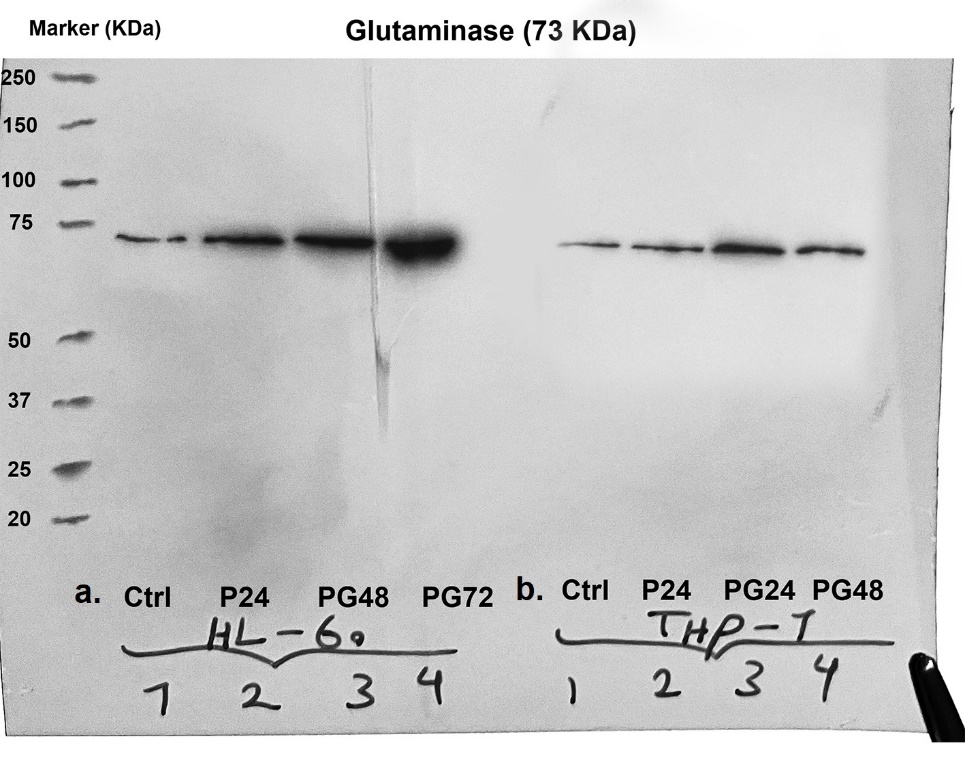


**Supplementary figure 3. The Glutaminase (GLS) protein expression in HL-60 and THP-1 cell lines.** (a.) Displays the full-length gels of GLS protein bands in control (ctrl), 24 hours after PMA treatment (P24), 48 hours (PG48) and 72 hours (PG72) after Gal-9 treatment groups in the HL-60 cell line. (b.) Presents the full-length gels of GLS protein bands in control (ctrl), 24 hours after PMA treatment (P24), 24 hours (PG24) and 48 hours (PG48) after Gal-9 treatment groups in the THP-1 cell line. Cropped 4 grouping blots are displayed in the Figure 4 (d.) in the HL-60 cell line and Figure 5 (d.) in the THP-1 cell line in the manuscript, respectively.
